# Mapping modifiable determinants of medication adherence in bipolar disorder (BD) to the theoretical domains framework (TDF): a systematic review

**DOI:** 10.1017/S0033291721001446

**Published:** 2021-05

**Authors:** Asta Ratna Prajapati, Alexandra Dima, George Mosa, Sion Scott, Fujian Song, Jonathan Wilson, Debi Bhattacharya

**Affiliations:** 1 Norfolk and Suffolk NHS Foundation NHS Trust, Norwich NR6 5BE, UK; 2 University of East Anglia, Norwich Research Park, Norwich NR4 7TJ, UK; 3 University of Lyon, Lyon, France; 4 Devon Partnership NHS Trust, UK

**Keywords:** Adherence, barriers and facilitators, bipolar disorder, compliance, theoretical Domains Framework

## Abstract

**Background:**

Around 40% of people with bipolar disorder (BD) are non-adherent to medication leading to relapse, hospitalisation and increased suicide risk. Limited progress in addressing non-adherence may be partly attributable to insufficient understanding of the modifiable determinants of adherence that require targeting in interventions. We synthesised the modifiable determinants of adherence in BD and map them to the theoretical domains framework (TDF).

**Method:**

We searched CINAHL, Cochrane Library, Embase, LILACS, Medline, PsychINFO and PubMed until February 2020. We included studies reporting modifiable determinants of adherence in BD. Two reviewers independently screened studies, assessed quality, extracted modifiable determinants and mapped them to TDF.

**Results:**

We included 57 studies involving 32 894 participants. Determinants reported by patients spanned 11 of the 14 TDF domains compared to six domains represented by clinician/researcher. The TDF domains most commonly represented (% and example) in studies were: ‘Environmental context and resources’ (63%, e.g. experiencing side effects), ‘Beliefs about consequences’ (63%, e.g. beliefs about medication effects), ‘Knowledge’ (40%, e.g. knowledge about disorder), ‘Social influences’ (33%, e.g. support from family/clinicians), ‘Memory, attention and decision processes’ (33%, e.g. forgetfulness), ‘Emotion’ (21%, e.g. fear of addiction) and ‘Intentions’ (21%, e.g. wanting alternative treatment). ‘Intentions’, ‘Memory, attention and decision processes’ and ‘Emotion’ domains were only reported by patients but not clinicians.

**Conclusions:**

Clinicians may be underappreciating the full range of modifiable determinants of adherence and thus not providing adherence support reflective of patients' needs. Reporting of modifiable determinants in behavioural terms facilitates developing theory-based interventions to address non-adherence in BD.

## Background

Bipolar disorder (BD) is generally a recurrent, lifelong mental health condition with a high risk of disability and excess mortality (Grande, Berk, Birmaher, & Vieta, 2016; Vazquez, Holtzman, Lolich, Ketter, & Baldessarini, 2015). The worldwide lifetime prevalence of the BD is estimated at 1% (Rowland & Marwaha, 2018). BD usually requires long-term medication but an estimated 40% of people are non-adherent to medication leading to relapse, functional impairment and suicidality (Gonzalez-Pinto et al., 2006; Lingam & Scott, 2002; Strakowski et al., 1998; Velligan et al., 2009). Medication non-adherence increases the probability of hospitalisation by at least five times (Scott & Pope, 2002).

Efforts to improve medication adherence have had marginal effects (Easthall, Taylor, & Bhattacharya, 2019; Nieuwlaat et al., 2014). This may be due to limited understanding of the modifiable determinants of medication adherence and existing support focussing on a narrow range of adherence determinants. We define modifiable determinants as ‘any determinants (barriers or facilitators) of medication adherence that can be modified by the patient, carer, or the prescriber within a short timeframe (days or weeks) to improve adherence’. We define a barrier as ‘a circumstance that prevents the patient from taking their medication as prescribed’, whereas a facilitator is ‘a circumstance that makes the process easy or easier’ (*Oxford English dictionary online*: Oxford university press, 2017). Some evidence syntheses report determinants of adherence to mental health treatment but they do not clearly distinguish between those that are modifiable, such as knowledge regarding how to take medication and non-modifiable such as age and gender. Such distinction is vital to allow adherence interventions to target modifiable determinants.

Furthermore, any differences between the perspective of clinicians and patients on determinants of medication adherence require exploration. Clinicians are the treatment experts but patients are the experts of their lived experience. Their goals, priorities and knowledge of the situation may differ. Thus, clinicians and patients may see the determinants of medication adherence differently (Devine, Edwards, & Feldman, 2018; Velligan et al., 2009). Exploring such differences will help design adherence support based on the patient's needs.

A recent systematic review by Garcia et al. provides an overview of barriers to medication adherence in BD and schizophrenia (Garcia et al., 2016). However, the study limited on determinants of adherence to antipsychotics (one group of medication to manage BD). Other common medications for BD are known as mood stabilisers which includes lithium. The omission of adherence determinants to lithium and other mood stabilisers is significant since lithium is recognised as the first-line gold standard long-term therapy in BD [Grunze et al., 2013; National Institute of Health and Care Excellence (NICE), 2014]. It is also noteworthy that the challenges to adhere to lithium may be different as lithium is a narrow therapeutic index drug and thus require a regular blood test, some dietary restrictions and has significant interactions with other medications [National Institute of Health and Care Excellence (NICE), 2014]. Furthermore, the review does not delineate modifiable from non-modifiable determinants which lack specific behaviour change techniques (BCTs) (Michie, Johnston, Francis, & Hardeman, 2008).

Additionally, the lack of behavioural theory underpinning the evidence synthesis in medication adherence in BD is evident. Thus, a systematic review of modifiable determinants of all treatment option in BD underpinned by theoretical framework is needed. Further details regarding the rationale for this systematic review are provided in the published protocol (Prajapati et al., 2019).

This systematic review aimed to identify modifiable determinants of medication adherence in BD reported in the literature and map them to the theoretical domains framework (TDF).

This study is a part of the Collaborative Medication Adherence in Bipolar disorder (C-MAB) project funded by Health Education England/National Institute for Health Research UK. The C-MAB project aims to develop a medication adherence tool for people with BD. The project advisory board includes stakeholders, patients, carers, clinicians, health psychologist and experts in behavioural medicine.

## Method

The study was registered with PROSPERO, registration number: CRD42018096306.

The protocol with detailed methods for this systematic review is published elsewhere (Prajapati et al., 2019), and a summary of the methods is provided below.

We searched CINAHL, Cochrane Library, Embase, LILACS, Medline, PsychINFO and PubMed from database inception to October 2018 using the search terms ‘Treatment Adherence and Compliance’, ‘Bipolar Disorder’ and ‘Psychotropic Drugs’. We updated the search in February 2020. The detailed search strategy is available in online Supplementary file.

We included primary, qualitative and quantitative studies published in the English language and studies explicitly reporting modifiable determinants of medication adherence in BD in adults. We excluded reviews, intervention studies to improve adherence, case reports, clinical guidelines or general disease management articles, studies involving short-term treatment of acute agitation or treatment other than medication such as psychotherapy.

Two reviewers (AP, DB, FS, GM, JW and SS) independently screened the study abstracts and full-texts and carried out the quality assessment. Disagreements were resolved through discussion and referral to a third reviewer for arbitration if necessary. A range of quality assessment tools (Center for Evidence Based Management, 2014; Critical Appraisal Skills Programme, 2018; National Institute of Health, 2014) was used according to the study designs (Frambach, van der Vleuten, & Durning, 2013).

We used Preferred Reporting Items for Systematic Reviews and Meta-Analyses (PRISMA) (Moher, Liberati, Tetzlaff, Altman, & The PRISMA Group, 2009) checklist for data extraction and reporting. The completed PRISMA checklist is available in online Supplementary file 2.

### Underpinning theoretical framework

We used framework analysis with the TDF as an *a priori* framework, to map modifiable determinants of medication adherence to their relevant TDF domain. The use of a theoretical framework provides a broad lens through which to capture the literature identified modifiable determinants. The TDF is a comprehensive framework capturing 33 theories and 84 theoretical constructs related to behaviour change (Atkins et al., 2017). Atkins et al. report the definition of each TDF domain and construct within each domain (Atkins et al., 2017). TDF was developed as a consensus framework by experts in health service research and behaviour science (Michie et al., 2005). The TDF offers the additional advantage that each of its 14 domains is coupled with BCTs (Michie et al., 2008). Thus, mapping modifiable determinants of adherence to the TDF offers a significant utility for intervention development.

Two independent reviewers (AP, AD, DB and SS), with experience in using the TDF, extracted modifiable determinants and coded them to the TDF domains using Nvivo 12 (QSR International Pty Ltd, 2018). For example, the extracted text ‘lack of awareness that medication needed to be taken regularly led to non-adherence’ in the study was coded to the TDF domain ‘Knowledge’. In addition to the 14 TDF domains, we also created another domain called ‘Others’ for any modifiable determinant not suitable to map to those 14 domains. Agreement between two reviewers in mapping modifiable determinants to the same TDF domain was calculated in SPSS version 25 using Cohen's kappa.

We grouped the modifiable determinants into overarching themes (Gale, Heath, Cameron, Rashid, & Redwood, 2013). We also coded whether the modifiable determinants were barriers or facilitators and whether it was reported by patients, clinicians, carers or any other third parties.

## Results

**Fig. 1.**
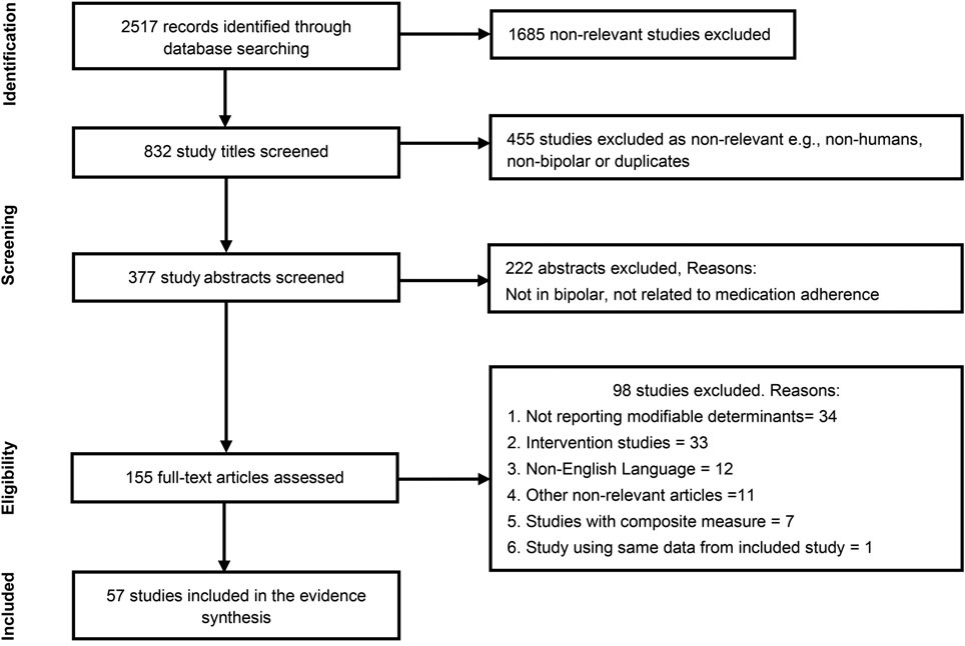
PRISMA flow diagram. PRISMA = Preferred Reporting Items for Systematic Reviews and Meta-Analyses.

From the 2517 studies retrieved, we included 57, comprising 32 894 patients and clinicians. Figure 1 provides the screening process, number of retrieved studies, number of studies included and excluded during title screening, abstract screening and full text screening as well as the reasons for exclusion. The primary reasons for exclusion at full-text screening were failure to report modifiable determinants or reporting an intervention to address adherence.

### Study characteristics

Summary characteristics such as study design, participant details and, country in which the included study was conducted are presented in Table 1. Fifty studies explored determinants from the perspective of patients and two (Vieta et al., 2012; Younas, Bradley, Holmes, Sud, & Maidment, 2016) from clinicians' perspective. Three studies included both patient and clinician perspectives (Baldessarini, Perry, & Pike, 2008; Maczka, Siwek, Skalski, Grabski, & Dudek, 2010; Pope & Scott, 2003). Further two studies were from the researcher's perspective (Gianfrancesco, Sajatovic, Tafesse, & Wang, 2009; Greene et al., 2018). However, none of the studies included carers. Most of the included studies collected data via surveys or interviews. The majority (79%) of the studies were conducted in the USA and Europe. A majority of the studies (64%) were focused purely on BD. Of the 57 included studies, 33% (Arvilommi et al., 2014; Baldessarini et al., 2008; Bauer et al., 2013; Fleck, Corey, Strakowski, & Keck, 2005; Grover, Ghosh, Sarkar, Chakrabarti, & Avasthi, 2014; Hajda et al., 2015; Johnson et al., 2007; Jonsdottir et al., 2013; Jose, Bhaduri, & Mathew, 2003; Manwani et al., 2007; Nagesh, Kishore, & Raveesh, 2016; Pope & Scott, 2003; Ralat, Depp, & Bernal, 2018; Roe, Goldblatt, Baloush-Klienman, Swarbrick, & Davidson, 2009; Scott & Pope, 2002; Stentzel et al., 2018; Vieta et al., 2012) explicitly focused on exploring barriers to adherence. Table 2 describes the quality of the included studies. The majority (65%) of the studies was of moderate quality, 19% were of high quality and 16% were of low quality.

**Table 1.** Summary of included studies

Study details	Study design	Study included	Study aims	No. of participants	Country	Non-adherence rate
Agyapong, Nwankwo, Bangaru, and Kirrane (2009)	Cross-sectional survey	BD, schizophrenia, depression	Assessment of associated factors that might influence compliance	409	Ireland	Not reported
Arvilommi et al. (2014)	Structured Clinical Interviews	BD only	Explored barriers of adherence: to investigate reasons were for treatment discontinuation	168	Finland	Not reported
Averous, Charbonnier, Lagouanelle-Simeoni, Dany, and Prosperi (2018)	Face to face interview	BD only	To explore associations between illness perceptions and adherence	38	France	Not reported
Baldessarini et al. (2008)	Survey	BD only	Explored barriers of adherence: risk factors to guide clinical prediction of nonadherence	429 patients + 131 psychiatrists	USA	33.8%
Bates, Whitehead, Bolge, and Kim (2010)	Web-based cross-sectional survey	BD only	To identify and describe correlates of medication adherence	1052	USA	49.5%
Bauer et al. (2013)	Naturalistic study where patient recorded their medication taking in self-reporting software	BD only	Explored barriers of adherence: to investigate regularity in the daily mood stabiliser dosage taken by patient and factors associated with irregularity	206	Germany	Not reported
Belzeaux et al. (2013)	Cross-sectional survey and interviews	BD only	To explore adherence behaviour and characterise the sociodemographic and clinical factors associated with adherence	382	France	25% of patients exhibited clear poor adherence
Bener, Dafeeah, and Salem (2013)	Survey	BD, schizophrenia, depression, anxiety and others	To examine the extent of compliance and non-compliance and examine the factors that affect compliance.	564	Qatar	41.8%
Clatworthy et al. (2007)	Semi-structured interviews	BD only	To explore in-depth beliefs about BD and its treatment that are associated with adherence to medication prescribed for BD	16	UK	8 reported non-adherence in the past and 5 reported current non-adherence
Clatworthy et al. (2009)	Questionnaire survey	BD only	The utility of the necessity concerns framework for understanding patient attitudes towards and levels of adherence	223	UK	30%
Col, Caykoylu, Karakas, and Ugurlu (2014)	Semi-structured interviews	BD only	To determine the factors affecting treatment compliance	78	Turkey	42.3%
Copeland et al. (2008)	Cross-sectional survey	BD only	To determine the association of insight and adherence	435	USA	27% had poor adherence based on missed dose and 46% had poor adherence based on Moriskey
Correard et al. (2017)	Cross-sectional observational	BD only	To investigate influence of age and neuropsychological functioning on adherence	353	France	47.3%
Darling et al. (2008)	Survey/interview	BD only	The influence of family and health stress, level of coping and internal health locus of control upon the life contentment of adherent and non-adherent individuals	100	USA	Not applicable as purposive sampling to include 50 adherent and 50 non-adherent patients
De Las, Penate, and Sanz (2014)	Survey	Bipolar, depression and dysthymia	To identify potential modelling factors influencing adherence	145	Spain	46.2%
De Las, Penate, and Cabrera (2016)	Survey	BD, schizophrenia, depression and others	To examine the role of perceived health control variables in psychiatric patients' adherence to prescribed treatment.	966	Spain	A quarter of patients self-reported a high level of adherence; 46.8% medium adherence and 28.2% a low adherence
Deegan (2005)	Interviews	BD, schizophrenia, major depression, and others	To understand how people with psychiatric disorders demonstrate the capacity for resilience in the ways they use or do not use psychiatric medications	29	USA	Not reported
Fleck et al. (2005)	Interviews	BD only	Explored barriers of adherence: to examine rates, self-perceived reasons and attitudes associated with non-adherence	50	USA	45% African American and 50% Whites totally non-adherent
Gianfrancesco et al. (2009)	Retrospective analysis of database	BD only	The study investigated monotherapy *v.* polypharmacy	3626	USA	Variable (depending on the medication and combination of medication)
Greene et al. (2018)	Retrospective analyses from database	Bipolar and schizophrenia (here we included only bipolar)	To compare differences in medication adherence and discontinuation between those who initiated a long acting injection and those who changed from one oral antipsychotic monotherapy to another	11 344	USA	61.1% in LAIs group and 78.5% in oral group
Greenhouse, Meyer, and Johnson (2000)	Survey	BD only	This report hypothesised that acceptance coping would correlate positively, and denial coping would correlate inversely with adherence	32	USA	75% of participants reported perfect adherence during the previous week
Grover et al. (2014)	Survey and semi structured interview with the patient and spouse/partner	BD only	Explored barriers of adherence: to evaluate the prevalence of sexual dysfunction in patients with BD receiving lithium and to study the correlates of sexual dysfunction	100	India	Varied (used BARS, MAQ) 84% took the prescribed doses of medications
Hajda et al. (2015)	Survey	BD only	Explored barriers of adherence: to determine the relationship between current adherence, medication discontinuing in the past and self-stigma	33	Czech Republic	Nineteen (57.6%) patients discontinued medication at least once in the past
Hibdye, Bekan, Dessalegne, Debero, and Sintayehu (2015)	Survey	BD only	Explored barriers of adherence: to assess the prevalence and factors associated with medication non-adherence among patients with BDs	410	Ethiopia	51.2%
Hou, Cleak, and Peveler (2010)	Survey	BD only	To investigate the impact of treatment and illness beliefs on medication adherence	35	UK	54.3% (probably non-adherent)
Inder, Lacey, and Crowe (2019)	Interviews	BD only	Analysis of medication adherence	36	New Zealand	NA
Johnson et al. (2007)	Survey	BD only	Explored barriers of adherence: to investigate factors associated with nonadherence and to assess the effect of patient preference on hypothetical medications	469	USA	23% always adherent, 37% usually adherent, 23% occasionally adherent, 17% rarely adherent
Jonsdottir et al. (2013)	Interviews	Bipolar and schizophrenia	Explored barriers of adherence: to investigate potential risk factors for medication nonadherence	255	Norway	13% Nonadherent, 31% partial adherent
Jose et al. (2003)	Survey	BD only	Explored barriers of adherence: to identify the reason for non-compliance	96	India	Not applicable (purposive sampling)
Kamaradova et al. (2016)	Survey	Bipolar, schizophrenia, depression, anxiety disorder and others	Explored barriers of adherence: to examine associations between self-stigma and adherence to treatment and discontinuation of medication in patients from various diagnostic groups	332	Czech Republic	124 patients (37.35%) admitted they had discontinued their medication previously
Keck et al. (1996)	Cohort study – patients evaluated at admission and followed up at 6 months	BD only	To identify clinical factors associated with maintenance antipsychotic treatment in patients with BD	77	USA	Varied, 41–68%
Keck, McElroy, Strakowski, Bourne, and West (1997)	Interviews	BD only	To assess patients' compliance with pharmacotherapy	140	USA	51%
Kraemer et al. (2013)	Observational study	BD only	To assess the duration of time on different mood stabilising medications and retention rates in standard clinical care	761	Germany	28.4%
Kutzelnigg et al. (2014)	Observational study	BD only	To determine factors associated with better compliance and to assess compliance between patients stabilised on olanzapine monotherapy and those stabilised on combination therapy	657	Austria, Romania, Hungary, Korea, Taiwan and Mexico	High levels of compliance (⩾80%) were observed in 67% of patients at baseline, increasing to 80% in study completers
Maczka et al. (2010)	Survey	Psychiatrists and patients with BD	An analysis and comparison of patients' and psychiatrists' beliefs regarding the most important aspects of BD treatment.	100 psychiatrists and 100 remitted patients	Poland	Not applicable
Manwani et al. (2007)	Structured Interviews	BD only	Explored barriers of adherence: to examine patterns and reasons of non-adherence	115	USA	17.5% in non-substance users, 34.5% in substance users
Morselli and Elgie (2003)	Survey	People with bipolar and non-bipolar (unipolar depression, or dysthymia or atypical depression)	To gain a better understanding of what it is like to live with BD	1732	Austria, Finland, France, Hungary, Holland, Italy, Portugal, Russia, Spain, Sweden and UK	47%
Nagesh et al. (2016)	An interviewer-assisted questionnaire-based study	Acute and transient psychotic disorder, borderline personality disorder, major depressive disorder, BD	Explored barriers of adherence: to assess the level of patients' adherence to psychotropic medications and to explore factors associated with non-adherence to medication	156	India	Adherence rate varied from low adherence (24.4%) through medium (34%) to high adherence (41.7%) among participants
Novick et al. (2017)	Post hoc analysis of 1-year observational study	Bipolar and schizophrenia	To explore non adherence with oro-dispersible *v.* standard normal tablet of olanzapine	903	France, Germany, Greece	Only reported average MARS scores
Perron, Zeber, Kilbourne, and Bauer (2009)	Survey	Bipolar, cyclothymia or schizoaffective disorder-bipolar subtype	To examine concurrent and predictive associations between provider support and adherence, access to care and health related quality of life	433	USA	Not reported
Pope and Scott (2003)	Survey	Patients with BD and their treating clinicians	Explored barriers of adherence: likely reasons for non-adherence identified by patients, the most common concerns of adherent and non- adherent subjects and the similarities and differences between clinicians' perceptions and patient concerns	72 patients taking lithium and 41 psychiatrists treating them	UK	46%
Ralat et al. (2018)	Focus group	BD only	Explored barriers of adherence: to identify patients' perspectives on the reasons for nonadherence to psychiatric medication	22	Puerto Rico	68% of participants reported nonadherence during the week of recruitment
Roe et al. (2009)	Semi-structured interviews	Bipolar and schizophrenia	Explored barriers of adherence: to explore why and how people with a serious mental illness choose to stop taking prescribed medication	7	Israel	Not applicable
Rosa et al. (2007)	Survey	BD only	To determine plasma and red blood cell lithium concentrations in bipolar patients at the same time as estimating attitudes and knowledge about lithium treatment in adherence scales	106	Brazil	33.06% based on MARS >7 14.4% based on plasma lithium
Rosenblat et al. (2018)	Survey	Bipolar depression and Major depressive disorder	Explore factors that impact treatment decisions	896	Canada	Bipolar depression and Major depressive disorder
Sajatovic, Bauer, Kilbourne, Vertrees, and Williford (2006)	Interview and self-report	BD only	Evaluated factors related to adherence	184	USA	38.6%
Sajatovic et al. (2009)	Interviews	BD only	This cross-sectional analysis examined clinical and subjective variables in relation to adherence	140	USA	19.3%
Sajatovic et al. (2011)	Interview plus quantitative assessments, adherence behaviour and treatment attitudes	BD only	This mixed-method analysis evaluated factors related to adherence among 20 poorly adherent community mental health clinic patients with BD	20	USA	Not applicable
Scott and Pope (2002)	Structured clinical interviews	BD (*n* = 78) and major depressive disorder (*n* = 20)	Explored barriers of adherence: to explore the prevalence and predictors of nonadherence with mood stabilisers	98	UK	Variable (47% had been non-adherent within last 2 years)
Sharma et al. (2012)	Survey	BD, schizophrenia and depression	This study examined the rates of medication non-adherence, associated disease, illness, treatment and physician-related factors of compliance	400	India	40.2%
Stentzel et al. (2018)	Interviews	BD, schizophrenia, schizotypal and delusional disorder, depression	Explored barriers of adherence: to examine potential determinants of non-adherence for patients with severe mental disorders	127	Germany	54% of the participants reported some kind of non-adherence
Teter et al. (2011)	Interviews	BD only	The study examined the impact of substance use disorder history with regards to medication-taking behaviours and attitudes	54	USA	Not reported
Vargas-Huicochea, Huicochea, Berlanga, and Fresan (2014)	Semi-structured interviews	BD only	To characterise the patients' perceptions and to give information that can help identify some of the factors involved in the treatment nonadherence	50	Mexico	Not reported
Vieta et al. (2012)	Survey	Psychiatrist treating bipolar patients	Explored barriers of adherence: to canvas the opinions of psychiatrists treating patients with BD and ascertain their perceptions of potential reasons for partial and non-adherence	2448	Austria, France, Germany, Italy, Spain, Switzerland, Turkey and UK	Psychiatrists estimated that 57% of their patients were partially or non-adherent
Weiss et al. (1998)	Structured interviews	Coexisting BD and substance use disorder	The study examined the pattern of medication compliance and reasons for non-compliance	44	USA	Variable and dependent on individual medication
Younas et al. (2016)	Interviews	Mental health pharmacists	To explore the views and experiences of UK mental health pharmacists regarding the use of shared decision making in antipsychotic prescribing in people with serious mental illness	13	UK	Not applicable
Zeber et al. (2008)	Survey	BD, cyclothymia, or schizoaffective disorder-bipolar subtype	The study examined the association between adherence and therapeutic environment perceptions among veterans with BD	435	USA	27%

**Table 2.** Quality of included studies

High quality (*n* = 11)	Moderate quality (*n* = 37)	Low quality (*n* = 9)
Belzeaux et al. (2013) Clatworthy et al. (2009) Copeland et al. (2008) Deegan (2005) De Las et al. (2016) Hou et al. (2010) Kamaradova et al. (2016) Sajatovic et al. (2006) Sajatovic et al. (2009) Sajatovic et al. (2011) Vargas-Huicochea et al. (2014)	Agyapong et al. (2009) Arvilommi et al. (2014) Averous et al. (2018) Baldessarini et al. (2008) Bates et al. (2010) Bauer et al. (2013) Clatworthy et al. (2007) Col et al. (2014) Darling et al. (2008) De Las et al. (2014) Fleck et al. (2005) Gianfrancesco et al. (2009) Greene et al. (2018) Grover et al. (2014) Hibdye et al. (2015) Inder et al. (2019) Johnson et al. (2007) Jonsdottir et al. (2013) Keck et al. (1996) Kraemer et al. (2013) Kutzelnigg et al. (2014) Maczka et al. (2010) Manwani et al. (2007) Morselli and Elgie (2003) Nagesh et al. (2016) Novick et al. (2017) Pope and Scott (2003) Ralat et al. (2018) Roe et al. (2009) Rosenblat et al. (2018) Scott and Pope (2002) Stentzel et al. (2018) Teter et al. (2011) Vieta et al. (2012) Weiss et al. (1998) Younas et al. (2016) Zeber et al. (2008)	Bener et al. (2013) Correard et al. (2017) Greenhouse et al. (2000) Hajda et al. (2015) Jose et al. (2003) Keck et al. (1997) Perron et al. (2009) Rosa et al. (2007) Sharma et al. (2012)

### Reported modifiable determinants of medication adherence

We extracted 290 modifiable determinants, which were grouped into 33 themes and mapped to 11 TDF domains. Inter-rater reliability for mapping the modifiable determinants to the TDF domains was 76% (Cohen's kappa 0.71), indicating substantial agreement between the reviewers (Landis & Koch, 1977). Cohen's kappa was calculated using SPSS 25.0 (IBM Corporation, 2017). Examples of the modifiable determinants, themes of determinants and TDF domains to which they were mapped are reported in Table 3.

**Table 3.** TDF domains, themes of determinants and examples of determinants (barriers and facilitators)

TDF domain (no. of studies reporting the domain)	Themes	Examples of determinants of medication adherence	
		Barriers	Facilitators
Environmental context and resources (*n* = 36)	Side effects of medication^a^	• Experience of actual side effects such as sedation, weight gain, sexual dysfunction, fatigue, cognitive impairment, extra-pyramidal and hormonal side effects	
	Medication formulation and treatment regimen	• Number and frequency of prescribed medication regimen with more complex/demanding regimens being negatively associated with adherence	• Long-acting injections had higher adherence than oral medications
	Ineffective medications^a^	• Medication not working or worsening symptoms after taking medication	
	Cost of medication	• Too expensive or inability to pay	• Free medication
	Irregular routine^a^	• Irregular daily routine or work schedule	
	Access to health care providers	• Poor access to mental health service including unavailability of doctors, difficulty getting transportation to appointments	
Belief about consequences (*n* = 36)	Belief about the necessity of medication either during treatment initiation or maintenance phase^a^	• Belief they ‘did not need’ medications for BD • ‘Felt well, saw no need to take medication’ • ‘If there are no symptoms, why take medications’	
	Belief about the positive or negative effects of medications^a^	• Felt less creative, less productive, less of myself, ‘missing highs’ • Concern about side effects	• Not wanting to be sick, to keep mood stable and functioning • The high belief that treatment would be helpful
	Doubt about the effectiveness of medication	• Belief that medication does not work	
	Belief that it is unnatural to take psychotropic medications	• Belief that it is unhealthy and unnatural to take medication to keep mood stable	
	To avoid punishment/trouble		• Belief that they will be sectioned or hospitalised if they did not take their medication
Knowledge (*n* = 23)	Knowledge about BD and its treatment^a^	• A lack of knowledge and awareness about the course of illness and treatment • Majority of the non-compliant patients were not aware that the Lithium stabilises the mood • Not knowing the need to take medication regularly • Not knowing that medications should be continued even when free of symptoms	• Better insight into illness • A high coherent understanding of their disorder • Being sufficiently informed about the disorder and its treatment • ‘My mental health care provider team made me aware of what to expect from good bipolar disorder care’
	Understanding how and when to take medication	• Unclear about prescription directions • Misunderstanding prescription directions	
Social influences (*n* = 19)	Personal support by the care provider		• ‘My mental health care provider team makes sure that we stay in regular contact’ • ‘I feel understood by my mental health care provider team’
	Feeling stigma	• The more self-stigmatised the patients were the lower their adherence • Ironically, as though the mental illness was not associated with enough stigma, the decision to cease medication, even when experienced as an important part of one's personal recovery, was stigmatising in its own right, leading them to conceal their decision and thus feel alone	
	Support or opposition from family, friends, relatives to diagnosis and treatment^a^	• Family and friends discouraging from taking medication • ‘My mother said I should think about it and try to use … reason and creativity instead of the medication’	• Having someone to support medication taking, monitoring symptoms, etc. • ‘I used medication to please my parents, who strongly supported it’
Memory, attention and decision process (*n* = 19)	Forgetfulness/carelessness	• Forgetting or not remembering to take the medication as prescribed • Laziness or careless at times about taking medications	• Individuals had a variety of methods to help them remember to take medications, including putting them in a consistent/specific place, labelling or writing reminders, taking medications at a consistent/specific time
	Medication taking routine	• Difficulties in maintaining pill-taking routines	· Attaching medication taking to other routine behaviours (e.g. taking medication after cleaning teeth)
Emotion (*n* = 12)	Fear of addiction or side effect of medication	• Worried about being dependent on medications • Fear of side effects of medications	
	Feeling threatened		• The threat of hospitalisation if medication is not taken
	Feeling of not being able to fulfil a social role	• Could not take care of my kids while on medication because I did not have the drive, or I just slept and slept on that medication	
	Negative feeling with medication prescribing and administration process	• Negative experience of how the medication was prescribed or administered • Taking medication every day is a frustration • Bothered that mood was controlled by medication	
Intentions (*n* = 12)	Denial of illness or illness severity	• Among reasons for non-adherence, denial of illness was the most commonly specified. With higher denial, adherence decreased exponentially	• Adherent patients tended to accept that they are ill
	Acceptance or denial of the need for treatment	• More than half of non-compliant patients expressed that they do not accept lithium treatment for a long time and as a normal routine	• From the compliant patients, there was 100% of acceptance of Lithium treatment
	Intentional non-adherence	• Not wanting to take medication • Wanting to take too much medication to get intoxicated	
Social, professional role and identity (*n* = 9)	Listening and shared decision making^a^	• Absence of shared decision making was believed to result in non-adherence and high rates of re-admission to hospitals	• ‘I feel that my health care practitioner has provided me choices and options about my health’
	Relationship with the prescriber		• Better patient-physician relationship • Satisfied with the competence of the doctor
	Being in control of the treatment regime	• Wanted to self-adjust the dose • Using regularly prescribed medication as ‘standby drugs' to stop mania	
Belief about capabilities (*n* = 6)	Belief in self and control^a^		• I think it's like any other condition … the more autonomy you give the patient … the more likely they are to comply
	Conflicting belief between clinician and patient	• Some research participants reported that clinicians interpreted their valued personal strengths and self-assessed health resources as part of psychopathology. Unsurprisingly, this led to non-adherence	
Goals (*n* = 3)	Different priorities over medication taking	• Psychiatric medications interfered with the things that give life meaning and purpose • Relief from personal stress was more important than taking medications	
	Desire to experience manic symptoms	• Stopping medication to experience mania	
Skills (*n* = 2)	Provision of training to manage BD		• ‘My mental health care provider team has provided training in what I need to do to carry out good bipolar disorder care’
Optimism (*n* = 0)	No determinants mapped to this domain		
Reinforcement (*n* = 0)	No determinants mapped to this domain		
Behavioural regulation (*n* = 0)	No determinants mapped to this domain		

a Clinicians only reported these themes of determinants.

Some facilitators were reported as the opposite of barriers. For example, ‘cost of medication’ was identified as a barrier in the ‘Environmental context and resources’ domain, for which ‘medication being free of charge’ represented the corresponding facilitator. In other cases, facilitators were occasionally worded as BCTs. For example, forgetfulness represented a barrier in the ‘memory, attention, and decision processes’ domain, for which the corresponding facilitators were reminders and formulating routines; these were classified in the BCT category of ‘prompts and cues’ which may successfully modify behaviour by addressing determinants in this TDF domain (Johnston et al., 2020).

The TDF domains represented in the greatest number of studies were ‘Environmental context and resources’ (63% of studies) and ‘Beliefs about consequences’ (63% of studies). Experience of side effects (49% of studies) and the nature of the medication, e.g. tablet, injection and dose frequency (22% of studies) were the main determinants mapped to the former; acting as barriers when unacceptable and facilitators when acceptable to patients. Beliefs about the likely positive/negative outcomes arising from adhering (36% of studies) and a belief that the medication is not needed (25% of studies) were the main determinants mapped to the latter.

Other TDF domains (and corresponding themes of determinants) reported in 20% or more studies, among all studies, were ‘Knowledge’ (whether the patient had sufficient knowledge about BD or its treatment); ‘Social influences’ (support or opposition from family, friends, relatives, clinicians regarding adherence); ‘Emotion’ (fear of addiction to or side effect from medication); ‘Memory, attention, and decision process’ (forgetfulness/carelessness with medication taking) and ‘Intentions’ (denial of illness or need for treatment).

Modifiable determinants were most frequently reported in the context of barriers rather than facilitators. However, unlike most other TDF domains, for ‘social influences’, facilitators and barriers were reported with similar frequency. This trend was also observed for ‘Social/Professional Role and identity’. Modifiable determinants related to ‘Goals’ and ‘Skills’ were infrequently reported. No determinants were mapped to the TDF domains of ‘Optimism’, ‘Reinforcement’ and ‘Behavioural regulation’.

### Determinants from the perspectives of patients and clinicians

**Fig. 2.**
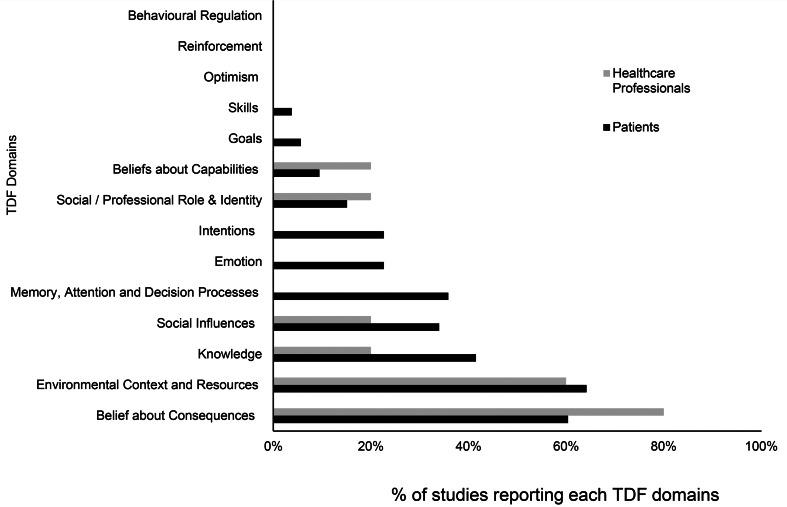
Comparison of TDF domains reported by patients and clinicians. No. of patients only studies = 50; no. of clinicians only studies = 2; no. of studies including patients and clinicians = 3. Two studies exploring researchers' perspectives were not included in this graph.

Figure 2 illustrates the TDF domains reported in patient studies compared to clinician studies. ‘Beliefs about consequences’ and ‘Environmental context and resources’ were the two most frequently reported TDF domains in both patient studies as well as clinicians studies. There were, however, noticeable differences in the range and nature of determinants reported by patients relative to clinicians. Determinants reported by clinicians were mapped to only six TDF domains compared to 11 TDF domains covered by patient studies. Only patient studies reported determinants which were mapped to the TDF domains ‘Intention’, ‘Memory, attention and decision process’ and ‘Emotion’. These domains included determinants such as denial of the illness or need for treatment, forgetfulness/carelessness and fear of addiction to or side effect of medication respectively (see Table 3 for more details).

Furthermore, ‘Goals’ and ‘Skills’ domains were reported in patient studies, albeit infrequently. An example of determinants in these two domains includes different priorities over medication taking and provision of training to manage BD, as shown in Table 3.

Clinicians reported modifiable determinants of adherence themed around lack of knowledge about medication, shared decision making, belief in self and perceived control, belief that medication is not needed, belief about positive or negative effects of medication, side effects, ineffective medication and irregular routine.

Two studies reported determinants from the researcher perspective (Gianfrancesco et al., 2009; Greene et al., 2018) namely medication formulations (such as tablets and injections) and the number of medications, both of which were mapped to ‘Environmental context and resources’ domain.

## Discussion

Synthesis of the literature through the theoretical lens of the TDF has enabled us to identify that negative emotions evoked by medication taking and intentional non-adherence make a notable contribution to non-adherent behaviour. In contrast to the focus of existing interventions on practical barriers to adherence (MacDonald, 2017; Torres-Robles et al., 2018), clinicians should additionally address negative emotions and lack of intentions.

In common with previous evidence syntheses, modifiable determinants were primarily barriers to adherence (Garcia et al., 2016; Velligan et al., 2009) with few reported facilitators. This may be an artefact of the included studies focussing on the challenges experienced by patients, rather than seeking to explore potential solutions. This hypothesis is supported by a third of the included studies explicitly seeking only barriers to medication adherence. For the few studies exploring facilitators, determinants that are not the opposite of barriers, such as wanting to keep the mood stable and not wanting to be hospitalised, have also been reported (Clatworthy, Parham, Horne, Bowskill, & Rank, 2007; Darling, Olmstead, Lund, & Fairclough, 2008). A strength of the present review is that we did not restrict the search to only adherence barriers; thus, we have identified a gap in the literature.

Current adherence interventions in BD focus mostly on education regarding medication and BD, cognitive therapy to address negative attitudes and beliefs, family therapy to encourage social support and technology to address forgetfulness (MacDonald, 2017; Torres-Robles et al., 2018). Furthermore, adherence support in the UK focusses on shared decision making regarding the choice of medication, side effects profile of medication, cost of medication and exploring patients beliefs [Care Quality Commission (CQC), 2018; National Institute of Healthand Care Excellence (NICE), 2009]. However, in this study, we found a broad range of other modifiable determinants that may be affecting medication adherence. This study provides clinicians with a comprehensive list of modifiable determinants of medication adherence, some of which are underappreciated by clinicians and unaddressed by existing adherence interventions.

### Advantages of mapping modifiable determinants to the TDF

Mapping determinants to the TDF allows them to be linked to BCTs. Thus, this study provides a foundation for developing a complex adherence intervention tailored to patients' needs based on their predominant determinants of adherence. The most frequently reported TDF domains of ‘Beliefs about consequences’ and ‘Environmental context and resources’ indicate that working with the patient's belief system, medication acceptability and tolerability are vital to support medication adherence. However, other modifiable determinants, particularly in ‘Intentions’, ‘Memory, attention and decision process’ and ‘Emotion’ domains, presented in this study may be equally or more relevant to individual patients. Thus, identifying the modifiable determinants most pertinent to an individual patient is critical to providing patient-centred adherence support.

The most frequently reported domain ‘Environmental context and resources’ was primarily related to medication characteristics such as side effects, treatment regime, medication effectiveness or cost of medication, etc. This finding accords with previous studies (Garcia et al., 2016; Kikkert et al., 2006; Salzmann-Erikson & Sjodin, 2018; Velligan et al., 2009). Side effects were represented in the domains of both ‘Environmental context and resources’ and ‘Beliefs about consequences’. This was because patients reported non-adherence arising from both experiencing side effects and being concerned that side effects may result from taking the medication. Each requires a different BCT, for example, the former may be better addressed by ‘restructuring the physical environment,’ e.g. by changing medication with a lower propensity of a particular side effect that the patient is experiencing. In contrast, the latter aligns with BCTs such as ‘pros and cons,’ e.g. discussing the risk and benefit of taking and not taking the medication (The UCL Centre for Behaviour Change, 2019).

The dominance of ‘Beliefs about consequences’ on medication adherence in this review is supported by other studies using the TDF (Crayton et al., 2017; Easthall et al., 2019). Belief about the necessity or concerns of medication were frequently reported determinants of adherence within this domain. As often reported in clinical practice, many people stop taking their medication once they feel better believing they no longer need them. On the contrary, some people believe they do not need medication at the start of the treatment and thus do not initiate them. Therefore, BCTs such as ‘pros and cons’ may play a vital role in medication adherence (The UCL Centre for Behaviour Change, 2019).

The absence of determinants mapped to the TDF domains ‘Optimism’, ‘Reinforcement’ and ‘Behavioural regulation’ does not necessarily mean that these three domains are unimportant to medication adherence in BD. Previous studies may not have explored these specific domains. Some adherence intervention studies suggest ‘Reinforcement’ using financial incentives may improve adherence (Priebe, Bremner, Lauber, Henderson, & Burns, 2016). Similarly, optimism, as measured by the revised Life Orientation Test (Herzberg, Glaesmer, & Hoyer, 2006), was reported to lead to improved adherence in acute coronary syndrome (Millstein et al., 2016). Revised Life Orientation Test includes statements such as ‘Overall, I expect more good things happen to me than bad’, ‘In uncertain times, I usually expect the best’ (Herzberg et al., 2006). However, these may not be modifiable. Future study should explicitly investigate the extent to which these unrepresented domains are relevant to non-adherence in this population and whether they are modifiable in the context of medication adherence.

Although there was a significant overlap between determinants reported by clinicians and patients, there were also notable distinctions. These distinctions may explain the limited progress made by clinicians in identifying and addressing non-adherence (Hartung et al., 2017; Nieuwlaat et al., 2014). However, these distinctions may also have arisen due to the small number of studies exploring the clinician's perspective.

Clinician reported determinants mapped to less than half of the TDF domains, suggesting that clinicians may not be aware of the broad range of determinants affecting medication adherence or studies were not designed to elicit this information from clinicians. The influence of negative emotion evoked by taking medication and intentional non-adherence was the most notable omission from clinicians' perspectives. This incomplete picture may result in adherence support poorly reflecting patients' needs (Brown et al., 2017). This is evident from current adherence support being focused on a very limited number of determinants (MacDonald, 2017; National Institute of Healthand Care Excellence (NICE), 2009; Thompson, Kulkarni, & Sergejew, 2000; Torres-Robles *et al*. 2018).

### Strengths and limitations

This study offers three novel aspects in the field of medication adherence research in BD. Firstly, the study focuses on potential adherence intervention targets by reporting only modifiable determinants. Secondly, as the application of theory is a core requirement for developing and implementing complex interventions, our use of a theoretical framework provides the foundations for developing future medication adherence interventions and their implementation. Finally, the comprehensive nature of a theoretical framework rather than an individual theory has enabled us to identify gaps in the literature.

Using the TDF as an *a priori* framework to organise modifiable determinants is a deductive approach. However, we did not constrain extraction of the determinants and mapping them to only the TDF domains as any determinants not aligned to the TDF would have been captured in the ‘Others’ category. The lack of detailed description of the determinants in some studies risked mapping them to incorrect TDF domains. For example, some studies described ‘hassle to acquire medication’ as a determinant of adherence. It could mean the patient has difficulty obtaining medication due to not knowing how to order their prescription or difficulty remembering to order a prescription or lack of transport/money/time to order prescription. Each interpretation would be mapped to a different TDF domain. Further qualitative study with patients will facilitate these further refinements in mapping.

We presented the modifiable determinants of adherence identified from a wide range of study designs. We recognise that the medium via which data are collected can influence the range of determinants captured. For example, interviews may elicit a greater range of determinants that are personal to the individual *v.* a structured survey of potentially relevant determinants (Lagard, Keegan, & Ward, 2003). This non-restrictive approach has contributed to identifying a list of modifiable determinants as comprehensively as possible which was one of the goals of this study.

### Implications for practice

We provide theory and evidence-based modifiable determinants that influence a patient's ability to adhere to their prescribed medication. All these determinants should, therefore, be considered and potentially discussed with patients when initiating treatment and at every review. Currently, clinicians may not be providing adherence support tailored to patients' wide-ranging needs.

### Implications for research

The application of a theoretical framework to the systematic review has enabled us to identify gaps in the literature where researchers have not sought to investigate the relevance of facilitators of adherence. Further research to explicitly capture the facilitators of adherence may help design future adherence interventions. The existing literature mostly represents the patient voice; absence of the carer voice is a notable gap given their role in supporting medication adherence in people with mental health problems (Deane, McAlpine, Byrne, Davis, & Mortimer, 2018). Future research exploring carers' views on modifiable determinants of medication adherence in BD is, therefore, needed.

## Data Availability

The data that support the findings of this study are available from the corresponding author, AP, upon a reasonable request.

## Supplementary material

For supplementary material accompanying this paper visit https://doi.org/10.1017/S0033291721001446. click here to view supplementary material
