# Supplementary material for: Mapping modifiable determinants of medication adherence in bipolar disorder (BD) to the theoretical domains framework (TDF): a systematic review
Source: Psychol Med. 2021 May 19;51(7):1082–98. doi: 10.1017/S0033291721001446 (PMC8188530; doi:10.1017/S0033291721001446)
Supplement: Supplementary file 1 [file S0033291721001446sup.zip › S0033291721001446sup001.docx]

**Supplementary File: Search Strategy**

| **Database** | **Search Strategy** |
| --- | --- |
| Pubmed | ((("Medication Adherence"[Mesh]) OR "Treatment Adherence and Compliance"[Mesh]) AND "Bipolar Disorder"[Mesh]) AND "Psychotropic Drugs"[Mesh] |
| Embase | ((exp "BIPOLAR DISORDER"/ AND exp "PATIENT COMPLIANCE"/) AND exp "PSYCHOTROPIC DRUG"/) |
| Medline | ((exp "BIPOLAR DISORDER"/ AND exp "TREATMENT ADHERENCE AND COMPLIANCE"/) AND (exp "PSYCHOTROPIC DRUGS"/ OR exp "ANTIMANIC AGENTS"/)) |
| CINAHL | ((exp "BIPOLAR DISORDER"/ AND exp "PATIENT COMPLIANCE"/) AND exp "MEDICATION COMPLIANCE"/) |
| Cochrane Library (CENTRAL) | #1MeSH descriptor: [Bipolar Disorder] explode all trees #2MeSH descriptor: [Psychotropic Drugs] explode all trees #3 MeSH descriptor: [Treatment Adherence and Compliance] explode all trees #1 AND #2 AND #3 |
| PsychINFO | ((exp "BIPOLAR DISORDER"/ AND exp "TREATMENT COMPLIANCE"/) AND (exp "NEUROLEPTIC DRUGS"/ OR exp "MOOD STABILIZERS"/)) |
| LiLACS | (tw:(bipolar disorder)) AND (tw:(treatment adherence AND compliance)) AND (tw:(psychotropic drug)) AND (instance:"regional") AND ( limit:("humans")) |
